# Effects of recombinant human growth hormone therapy on carbohydrate, lipid and protein metabolisms of children with Turner syndrome

**DOI:** 10.12669/pjms.304.4546

**Published:** 2014

**Authors:** Weibin Qi, Shuxian Li, Qiong Shen, Xiuxia Guo, Huijuan Rong

**Affiliations:** 1 Weibin Qi, Department of Neonatology, The Fourth Hospital of Shijiazhuang City, Shijiazhuang 050011, China.; 2 Shuxian Li, President's Office, The Fourth Hospital of Shijiazhuang City, Shijiazhuang 050011, China.; 3 Qiong Shen, Department of Gynecology and Obstetrics, Hebei Armed Police Corps Hospital, Shijiazhuang 050081, China.; 4 Xiuxia Guo, Department of Neonatology, The Fourth Hospital of Shijiazhuang City, Shijiazhuang 050011, China.; 5 Huijuan Rong, Department of Nursing, The Fourth Hospital of Shijiazhuang City, Shijiazhuang 050011, China.

**Keywords:** Growth hormone, Turner syndrome, Carbohydrate metabolism, Lipid metabolism, Protein metabolism

## Abstract

***Objective:*** To study the effect of recombinant human growth hormone (rhGH) therapy on carbohydrate, lipid and protein metabolisms of Turner syndrome (TS).

***Metho*** ***d*** ***s:*** Total 45 patients with TS admitted between Jul. 2008 and Jun. 2011 were involved in this study. All patients received the clinical evaluation of body fat, plasma lipids, proteins and oral glucose tolerance test (OGTT) before and after rhGH therapy.

***Results*** ***:*** Our results indicated a significant decrease of body fat (FAT%) from 23.56±4.21 to 18.71±2.23 but no obvious change on the level of fat mass (FM) (p>0.05) was observed after rhGH therapy. We also detected significant changes on plasma high-density lipoprotein cholesterol (HDL-C) from (1.65±0.58 mmol/L) to (2.20±0.65 mmol/L) and low-density lipoprotein cholesterol (LDH-C) from (2.55±0.55 mmol/L) to (2.10±0.54 mmol/L) after rhGH exposure. However, no statistical significance was detected on the level of plasma triglyceride (TG), cholesterol (CHO). Interestingly, the levels of plasma retinol binding protein (RbP) (32.55±4.28mg/L), transferrin (TRF) (2.95±0.40 mg/L), serum albumin (PRE) (250.00±45.50 mg/L) and albumin (propagated) (33.58±4.25 mg/L) were significantly increased. When it goes to the oral glucose tolerance test (OGTT) test, there were 10 impaired glucose tolerance (IGT) cases among all patients before and after rhGH therapy. No significant change was observed on homeostasis model assessment- insulin resistance (HOMA-IR) level during rhGH intervention.

***Conclusion*** ***:*** Abnormal lipid and protein metabolisms of the children with TS can be improved with rhGH therapy for 6 months.

## INTRODUCTION

Turner syndrome (TS) is characterized with abnormal chromosome type in which all or part of one of the sex chromosomes is absent or has other abnormalities.^1^ It is one of the most common genetic disorders, affecting approximately one in every 2,000 live-born females.^2^^,^^3^ Girls with TS typically experience gonadal dysfunction and short stature. And the affected individuals suffer from abnormal metabolisms of carbohydrate, lipid and protein as well. As a result, individuals with TS might develop type II diabetes four times higher than normal counterparts and be two times higher to develop type I diabetes.^4^^,^^5^ On the other hand, metabolic disorders are the leading cause of insulin resistance (IR), which in turn increase the risk of morbidity and disability.^6^

Recently, in order to minimize the symptoms, the treatment of TS is mainly focused on the recombinant human growth hormone (rhGF) therapy, which was reported to play important roles in carbohydrate accumulation, lipid decline and facilitating protein synthesis. In our study, we compared the differences of carbohydrate, lipid and protein metabolisms with or without rhGH therapy to reveal the clinical clue of the hormone replacement therapy.

## METHODS

***Subjects*** ***:*** A total of 45 girls karyotype-proven TS in the department of pediatrics of the Fourth Hospital of Shijiazhuang City were included in this study. Patients aged from 8 to 15 years (9.25±2.85yr) (bone age 8.75±3.25yr) were (2.50±0.58yr) fall behind the actual age. Patients weighted from 21 to 35 kg (28.12±7.85 kg) and heighted from 106 to 141 cm (124.15±18.50 cm). Clinical criteria for inclusion was as follows: a. short stature and failure to develop properly (<5cm/year); b. abnormal serum gonadotropin; c. backward skeletal age; d. X chromosome aneuploidy: 45 XO or variant of X-chromosome abnormality; e. clinical signs and symptoms included shield shaped thorax of heart, webbed neck and reproductive sterility. This study was conducted in accordance with the declaration of Helsinki after approval from the Ethics Committee of the Fourth Hospital of Shijiazhuang City. Written informed consent was obtained from all participants.

***Clinical evaluation*** ***:*** All patients underwent general clinical evaluations before and after being treated with rhGH for 6 months. Body fat detection included weight, Fat%, fat mass (FM) and body mass index (BMI). In addition, the level of patients’ plasma lipids, high-density lipoprotein cholesterol (HDL-C), low-density lipoprotein cholesterol (LDH-C), triglycerides (TG) and cholesterol (CHO) were evaluated as well. Oral glucose tolerance test (OGTT) test was conducted to evaluate the ability of patients’ glucose tolerance. In detail, patients used 1.75g/kg glucose solution with empty stomachs. Blood samples were collected at 0min, 30min, 60min and 120min after glucose consumption. In order to describe glucose regulation feedback within the patients, insulin resistance was measured by homeostasis model assessment (HOMA) method (employs fasting insulin and glucose levels) and was presented as HOMA insulin resistance index (HOMA-IR). In our study HOMA-IR was calculated as [Plasma glucose (GLU, mmol/L)*serum insulin (mIU/L)]/22.5. Plasma retinol binding protein (RbP), transferrin (TRF), serum albumin (PRE) and albumin (ALB) were quantified to evaluate the level of protein metabolism.

***Clinical treatment measures*** ***:*** All patients received rhGH (0.05mg/Kg*day) for at least 6 months. In addition to hormone therapy, patients were assigned to increase practice, balance diets and take good rest.

***Statistical analysis*** ***:*** All the data were analyzed by SPSS17.0 software (SPSS Inc, Chicago, IL, USA). Data presented as mean ±SD. Student *t* test was used to compare the variances before and after treatment. In all statistical analyses, a two-tailed *p* value ≤0.05 was considered statistically significant.

## RESULTS

***Quantification of patients’ body fat*** ***:*** To evaluate the lipid metabolism before and after rhGH therapy, patients’ FAT%, FM and BMI were tested separately. Interestingly, when patients were exposed with rhGH treatment, FAT% was decreased statistically (18.71±2.23) compared with the level before the treatment (23.56±4.21) (Table-I). However, no statistical significance has been detected on the levels of FM and BMI before (10.15±1.92kg), (19.25±3.40 kg/m^2^) and after (9.28±1.20kg) (18.90±2.20 kg/m^2^) the treatment (Table-I).

***Quantification of patients’ blood lipid level*** ***:*** To further evaluate the lipid metabolic efficiency of the patients’ who were treated with rhGH, plasma level of high-density lipoprotein cholesterol (HDL-C), low-density lipoprotein cholesterol (LDH-C), triglycerides (TG) and cholesterol (CHO) were measured. Compared to the plasma lipid levels before treatment, there were significant changes on the levels of plasma HDL-C from (1.65±0.58 mmol/L) to (2.20±0.65 mmol/L) and LDH-C from (2.55±0.55 mmol/L) to (2.10±0.54 mmol/L) after the exposure of rhHG. However, there was no significant change on the levels of TG and CHO detected (Table-II).

***Quantification of patients’ plasma proteins*** ***:*** To evaluate the protein metabolism before and after rhGH therapy, plasma levels of retinol binding protein (RbP), transferrin (TRF), serum albumin (PRE) and albumin (ALB) were tested. Interestingly, our study revealed significant increases on the levels of plasma RbP from (18.85±3.25 mg/L) to (32.55±4.28mg/L), TRF from (1.25±0.30 mg/L) to (2.95±0.40 mg/L), PRE from (103.00±52.50 mg/L) to (250.00±45.50 mg/L) and ALB from (21.25±2.28 mg/L) to (33.58±4.25 mg/L) after the rhGH therapy (Table-III).

**Fig.1:**
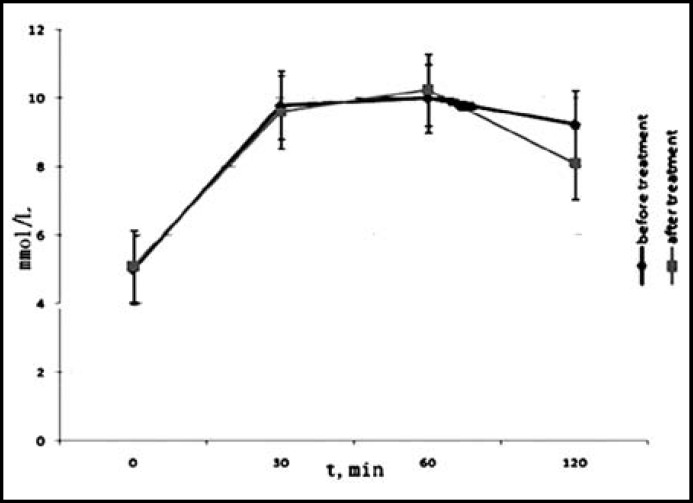
Level of blood sugar on different time points of OGTT test

**Fig.2:**
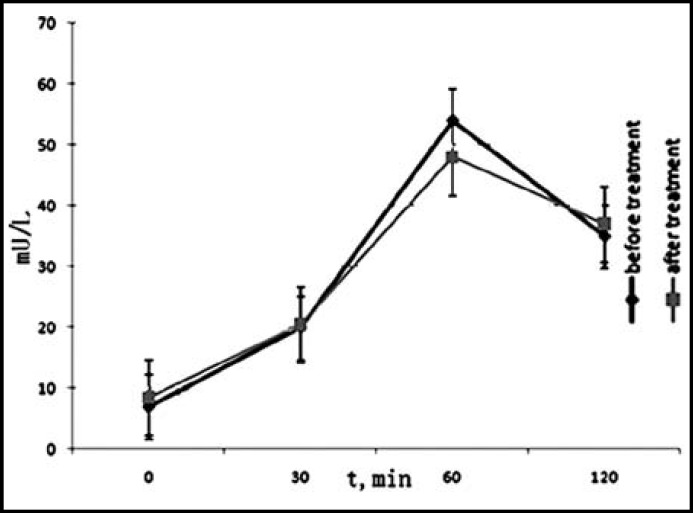
Level of blood insulin on different time points of OGTT test

***Analysis of*** ***o*** ***ral glucose tolerance test (OGTT)*** ***:*** There were 10 patients suffering from impaired glucose tolerance (IGT) before rhGH therapy. The cases of IGT were the same after the treatment. To evaluate the efficiency of patients’ glucose tolerance, we conducted the OGTT test on all the patients. Our data showed no statistical significance on either the level of blood sugar (Fig.1) or insulin (Fig.2). In addition, the HOMA-IR before and after rhGH therapy was 1.65±0.51 and 1.61±0.42, respectively. There was no statistical significance revealed in our current study.

## DISCUSSION

It was reported that there was lipid deposition in the individuals with TS who have lost weight due to the disease.^7^^,^^8^ The current study revealed a high level of body fat in patients with TS before being treated with rhGH, indicating a lipid accumulation caused by abnormal lipid metabolism. In the blood lipid level tests, we also detected high level of plasma TC, CHO and LDH-C and low level of HDL-C in patients before rhGh therapy. As reported by Judith et al.^9^ the variations of plasma TC, LDH-C and HDH-C correlated with patients’ age apparently, since girls during puberty would experience more opportunities of abnormal lipid metabolism. But there was no correlation between the change of CHO level and patients’ age, BMI and abnormality of chromosome. Our results showed obvious improvement on lipid metabolism of patients who underwent rhGH therapy. The patients’ body fat (Fat%) decreased significantly after rhGH therapy. In addition, in this current study we detected significantly increased plasma HDL-C and decreased plasma LDL-C as well. Furthermore, there were mild decrease on plasma TG and CHO though. We hypothesized that rhGH might accelerate both the decrease of body fat and the oxidation of lipid acid.^10^ There are two essential pass ways through which the rhGH might work on the lipid metabolism. On one hand rhGH could induce the receptor of adrenaline in fat cells to evoke the lipolytic effects.^11^ On the other hand, it prompts lipase to accelerate the lipolysis as well. GH may play an important role in the lipid metabolism of the body.^11^^,^^12^ Therefore, GH replacement therapy could somehow modify the abnormality of lipid metabolism.

**Table-I:** Patients’ body fat level

	***n***	***FAT(%)***	***FM (kg)***	***BMI (kg/m*** ^2^ ***)***
Before treatment	45	23.56±4.21	10.15±1.92	19.25±3.40
After treatment	45	18.71±2.23	9.28±1.20	18.90±2.20
p-value		0.023	0.553	0.610

**Table-II:** Patients’ blood lipid level

	***n***	***HDL-C(mmol/L)***	***LDL-C(mmol/L)***	***TG(mmol/L)***	***CHO(mmol/L)***
Before treatment	45	1.65±0.58	2.55±0.55	1.18±0.90	4.20±0.88
After treatment	45	2.20±0.65	2.10±0.54	0.95±0.48	4.05±0.50
p-value		0.032	0.040	0.103	0.923

**Table-III:** Patients’ plasma protein level

	***n***	***RbP(mg/L)***	***PRE(mg/L)***	***TRF(mg/L)***	***ALB(mg/L)***
Before treatment	45	18.85±3.25	103.00±52.50	1.25±0.30	21.25±2.28
After treatment	45	32.55±4.28	250.00±45.50	2.95±0.40	33.58±4.25
p-value		0.003	0.001	0.005	0.004

Under the general circumstances, GH mainly enhances the synthesis of protein or, in another word, restricts the degradation of protein as well as the amino acid in muscles. However, it could balance the protein metabolism much better when the body was involved in stress or other pathogenic circumstances.^13^ Our study detected the lower level of plasma RbP, TRF, PRE and ALB in patients before rhGH therapy, which may due to the enhanced lipolysis and synthesis of urea nitrogen when patients were in a long term GH shortage.^14^ As reported by Binnerts et al.^15^ there was an obvious increase in protein synthesis but no change took place in lipolysis when patients were exposed to rhGH therapy for the first month. And a delayed lipolysis has been detected when the treatment prolonged for 6 to 9 months. In this study, we detected the increased level of plasma RbP, TRF, PRE and ALB in patients who underwent the rhGH therapy for 6 months, which were in accordance with the previous reports.^15^ We hypothesized that GH might favor the uptake of amino acid by transporter.

Previous literatures revealed that the incidence of IGT in girls with TS was as high as 10% to 34%, which is 2-4 times higher than normal individuals.^16^ In our study, there were 10 girls (n=45) with TS suffering from IGT and the HOMA-IR was 1.65±0.51 before taking the rhGH therapy. It was suggested that TS patients bearing a high opportunity of abnormal glucose metabolism. Insulin resistance might restrict the pathway of glucose metabolism in cells, and weaken it’s sensitivity to glucose as well.^17^ Unfortunately, in our study, no significance on the HOMA-IR was detected, suggesting rhGH might make it even better other than getting the glucose metabolism worse. Not only could GH decrease glucose utilization and in turn increase the level of blood sugar, but also it acts insulin-like function, increasing the secretion of insulin by enhancing the stimulation of arginine and glucose to pancreas.^18^

In conclusion, patients with TS suffer from abnormal carbohydrate, lipid and protein metabolisms. Our data revealed that treatment with rhGH could improve the efficiency of lipolysis, maintain the balance of protein metabolism and to a mild extent better the metabolism of glucose.

## Authors contribution:

**Qiong Shen** **,** **Weibin Qi: **Conception and design of study.

**Weibin Qi, Shuxian Li, Qiong Shen** **: **Acquisition of data.

**Xiuxia Guo, Huijuan Rong** **: **Analysis and/or interpretation of data.

**Weibin Qi, Shuxian Li, Qiong Shen: **Drafting the manuscript.

**Qiong Shen** **: **Revising the manuscript critically for important intellectual content.
